# A usability study of a multicomponent video game-based training for older adults

**DOI:** 10.1186/s11556-019-0233-2

**Published:** 2020-01-11

**Authors:** Manuela Adcock, Floriana Sonder, Alexandra Schättin, Federico Gennaro, Eling D. de Bruin

**Affiliations:** 1 Department of Health Sciences and Technology, Institute of Human Movement Sciences and Sport, ETH Zurich, Leopold-Ruzicka-Weg 4, 8093 Zurich, Switzerland; 2 Department of Neurobiology, Care Sciences and Society, Karolinska Institute, Alfred Nobels Alle 23, 14183 Huddinge, Sweden

**Keywords:** Physical-cognitive intervention, Exergame, Healthy aging, Fall prevention, Elderly, Older adults, Usability, Game experience

## Abstract

**Background:**

Aging is often accompanied by a decline in sensory, motor and cognitive functions. These age- and lifestyle-related impairments may lead to reduced daily life functioning including gait disturbances, falling and injuries. Most daily life activities, e.g. walking, are tasks which require the concurrent interplay of physical and cognitive functions. Promising options for combined physical-cognitive training are video game-based physical exercises, so-called exergames. This study aimed to [i] determine the usability of a newly developed multicomponent exergame and [ii] explore its effects on physical functions, cognition and cortical activity.

**Methods:**

Twenty-one healthy and independently living older adults were included (10 female, 71.4 ± 5.8 years, range: 65–91) and performed 21 training sessions (each 40 min) over seven weeks. The multicomponent exergame included strength and balance training with Tai Chi-inspired and dance exercises. Participants rated the usability of the exergame (System Usability Scale) and reported on their emotional experience (Game Experience Questionnaire). Attendance and attrition rates were calculated to determine training compliance. Before and after the intervention, physical and cognitive functions as well as resting state electroencephalography (EEG) were assessed.

**Results:**

Results showed a high training attendance rate (87.1%, 18/21 training sessions on average) and a low attrition rate (9.5%, 2 drop-outs). System usability was rated high with a mean score of 75/100. Affective game experience was rated favorable. Gait speed under dual-task condition, lower extremity muscle strength and reaction times in a cognitive task (divided attention) showed significant improvements (*p* < .05). No significant pre-post differences were found for resting state EEG.

**Conclusions:**

The newly developed exergame seems usable for healthy older adults. Nevertheless, some aspects of the exergame prototype can and should be improved. The training showed to positively influence physical and cognitive functions in a small convenience sample. Future trials are warranted which evaluate the feasibility and usability of the exergame training in a more “real-life” in-home setting and assess the behavioral and neuroplastic changes in a larger population after a longer training period with comparison to a control group.

## Background

Age- and lifestyle-associated degenerative changes cause reduced daily life functioning including gait impairments and a higher fall risk in elderly [1]. Falling can lead to injuries, movement restrictions, loss of independence, social isolation, depression and a general decrease in well-being and quality of life [2]. Current numbers demonstrate that one out of three people aged 65 years and older fall annually and 20–30% of falls result in injury and hospitalization [3, 4]. Considering the significant impact on the individual lives of the growing elderly population as well as on healthcare costs, a strong need exists to examine interventions that aim to support healthy aging and prevent falls. For successful falls prevention, causes and risk factors of falling must be targeted. It is well known that age-related declines in sensorimotor systems lead to muscle weakness, reduced muscle strength and increased balance problems and, therefore, to gait disturbances as well as a higher risk of falling [5, 6]. Exercise interventions, which aim to improve physical functions such as strength or balance training, showed to reduce fall rates and risks [7–9].

Age-associated changes occur also on neuronal level. The aging brain exhibits structural changes in gray and white matter [10–12] and functional changes in cortical oscillatory activity patterns [13]. An age-related “slowing” in brain activity is described (with an increase in slow frequency ranges and a decrease in higher frequencies e.g. alpha frequency) [13, 14]. On a cellular level, physiological aging is characterized by a loss of synaptic contacts and an apoptosis of neurons which can lead to a decline of sensorimotor and cognitive functions [13]. Most human daily life activities, including walking, require physical and cognitive resources. Safe and stable walking, especially in a demanding environment, is based on intact continuous interactions of physical and cognitive functions [15–19]. With age-related changes affecting the whole system from brain to muscles, the performance of physical-cognitive activities is impaired which, in turn, is considered a main risk factor for falling in older adults [20–22]. Therefore, a combined physical-cognitive training is important for effective fall prevention [23, 24]. Regular physical activity in older age effects gait stability, health status and general well-being [25–27]. However, in most existing training approaches for fall prevention, no explicit attention is paid to cognitive functions and the physical-cognitive interplay. A promising option for simultaneous training of physical and cognitive functions are interactive video game-based physical exercises or so called exergames [28].

Exergames are defined as “any types of video games that require the player to be physically active and move to play the game” [29]. Due to a combination of physical exercises with cognitive stimulation, exergame training might be closer to daily life requirements. Another advantage of exergames may relate to the “gamification” approach of training. Several studies demonstrated that exergames have a high motivational potential by providing enjoyable gameplay [30, 31]. Moreover, exergame training can be applied in diverse settings (e.g. home-based training). Purpose developed exergames for public health and disease prevention are well advised to be designed according to target users’ expectations and needs [29]. Following a user-centered design approach, acceptability and training adherence seem to increase and this, in turn, can enhance specific training effects [32, 33].

Considering older adults’ needs and requirements, the Active@Home project, an international project of the Ambient Assisted Living Association, developed an exergame prototype by incorporating theoretical background from human movement sciences and neuropsychology and the art of game design. The Active@Home exergame enables multicomponent training including strength, balance and cognitive training components and focuses on supporting healthy aging including fall prevention. Before conducting full-scale studies, the usability of this newly developed exergame as well as its acceptance by older adults should be tested [34]. Especially for training interventions, a usable solution for the target population is mandatory to ensure training compliance and benefits. Therefore, the primary aim of this study was to determine the usability of the newly developed Active@Home exergame. The secondary aim was to explore whether the exergame intervention can influence physical and/or cognitive functions as well as cortical activity at rest. We hypothesized a high usability of the newly developed exergame training for older adults due to following a user-centered development and design approach and based on findings from literature showing a high usability of virtual reality and technology-based training for the older population [32, 35]. Moreover, positive effects on gait parameters, balance, functional muscle strength as well as attentional and executive functions and brain activity in alpha frequency have been hypothesized.

## Methods

### Study design and participants

Campbell and colleagues recommend an iterative phased approach starting with exploratory trials (phase II studies) before conducting definitive randomized controlled trials (phase III studies) [34]. This phase II exploratory study used a single arm pre-post testing design. From March to May 2017, potential participants were recruited through public advertisements in local newspapers and from the pensioner community ETH Zurich (PVETH, Switzerland). Assessments and intervention were performed at ETH Hönggerberg (Zurich, Switzerland). Measurements were conducted before (June 2017) and after (September 2017) the intervention period. In addition, a between-measurement consisting of two questionnaires was performed after the first week of training. Before the intervention period started, participants wore an activity monitoring device (StepWatch) for 1 week. The ETH Zurich Ethics Committee (Zurich, Switzerland) granted ethical approval (protocol number EK 2017-N-06). All participants were fully informed prior to participation and signed an informed consent form according to the Declaration of Helsinki before conducting any measurement.

The potential participants were screened using the Montreal Cognitive Assessment (MOCA) to assess cognitive status. Furthermore, the participants completed a health questionnaire including anthropometric data and questions about their health, medical history and physical activity level. Participants fulfilling all of the following inclusion criteria were eligible for the study: (1) age ≥ 65 years, (2) living independently, (3) healthy (self-reported), (4) able to walk at least 20 m with or without walking aids. Participants exhibiting at least one of the following criteria were excluded from the study: (1) mobility impairments that prevent from training participation, (2) severe and uncontrolled health problems (e.g. recent cardiac infarction, uncontrolled diabetes or hypertension), (3) orthopaedic disease that prevents from training participation, (4) neurological disease (e.g. history of stroke or epilepsy, Parkinson’s disease), (5) Alzheimer disease or other forms of dementia, (6) acute severe, rapidly progressive or terminal illness, (7) cognitive impairments (MOCA < 26 points), (8) intake of any psychoactive substances (e.g. neuroleptics, antidepressants), (9) high alcohol, caffeine or nicotine consumption. The minimal intended study sample size of 20 participants was based on previously conducted feasibility and usability studies [35, 36] and on a practitioner’s guide [37].

### Exergame intervention

From June to September 2017, the participants performed three training sessions per week for 7 weeks resulting in a maximum of 21 training sessions. The training sessions were scheduled individually from Monday to Friday with a guideline of no more than one training session per day. The 21 training sessions were distributed within a period of seven to 9 weeks as a maximum of 2 weeks holiday interruption was allowed in between. Each session consisted of 40 min training with the newly developed Active@Home exergame prototype including Tai Chi-inspired training (20–30 min) and dance exercises (10 min). Tai Chi-inspired exercises were a combination of lower-limb and core strength exercises and Tai Chi elements in three different stance positions (squat, plié, and lunge). Tai Chi-like movements were used as this ancient Chinese physical activity is often performed in a semi-squat posture, placing load on the lower limbs and core muscles [38]. These muscles are important for functional movements as walking [39, 40] and are positively influenced by Tai Chi training in the elderly [41]. Beside of increasing muscle strength, Tai chi has been shown to enhance balance and coordinative skills as well as cognitive functions; the later may be due to the cognitively demanding exercises [38, 42, 43]. To ensure optimal training effects, muscle loading recommendations for older adults were applied to the Tai Chi-inspired exercises (e.g. time under tension of 6 s per repetition, a rest of 4 s between repetitions, 7–9 repetitions per set, a rest of 60s between sets, a training volume of 2–3 sets per exercise) [44]. Additionally, dance exercises were included in the Active@Home exergame. Dancing exercises were based on common dances as Bachata, Disco Fox, Salsa, Waltz, Cha-Cha-Cha, and Jive and, in general, require motor components of balance, coordination, and agility, but also cognitive resources [45–47]. Dancing and the execution of rapid and well-directed steps has been shown to improve balance, coordinative skills, endurance and cognitive functions [48–53]. Both, Tai Chi and dancing are “holistic” and task-oriented physical activities [54, 55]. The exercises were accentuated with background music [56].

The exergame prototype implemented some basic training principles [57] as a feedback system with a real-time colour code for performance (red colour for bad performance, orange colour for moderate performance, green colour for good performance) and performance scores during and after each exercise. To ensure optimal challenge (optimal load of task demands) and increasing difficulty (progression), several difficulty levels for Tai Chi-inspired and dance exercises were developed. Progression was reached through more complex movements in the Tai Chi-inspired exercises (e.g. additional arm movements, upper body rotations, increased range of motion, longer time in unstable position) and through additional weights (e.g. filled water bottles), while faster and more complex motion sequences were performed in dance exercises.

**Fig. 1:**
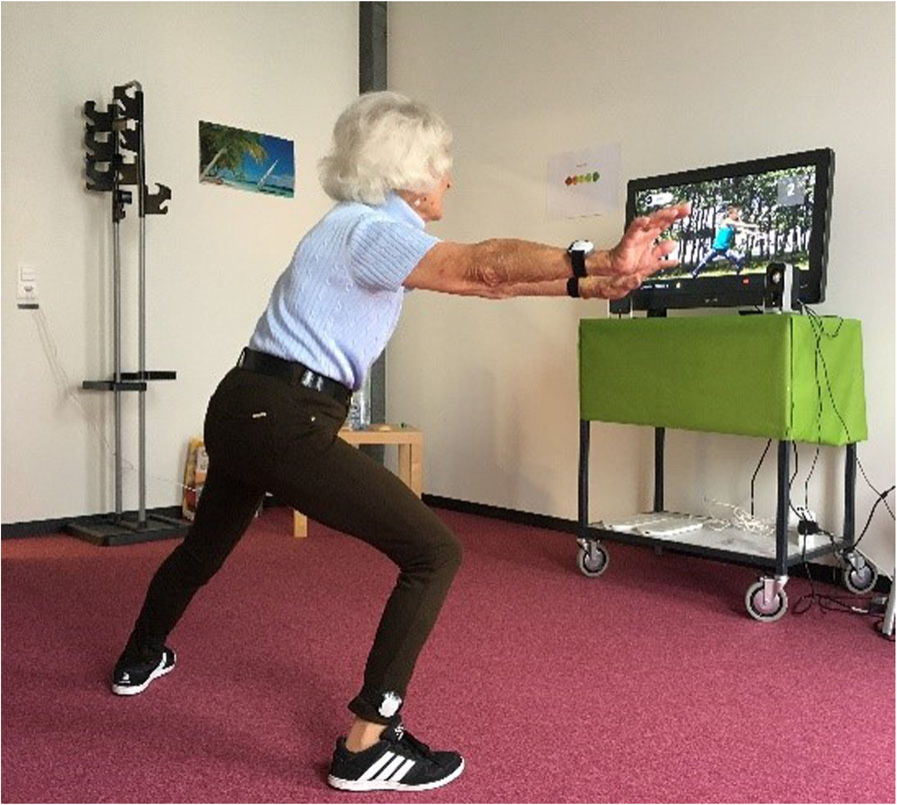
Set up of the Active@Home exergame training. Participants wore four IMUs at wrists and ankles for movement evaluation. On the TV screen, a virtual instructor presented the training exercise which had to be imitated by the participant. Written consent was obtained from the participant in the picture allowing the usage of the picture for scientific publication

The game story was about travelling in Europe and to train in several different European cities. To demonstrate the exercises, a virtual instructor was used. The game interface was presented on a TV screen connected to a laptop running the exergame software. For movement evaluation, the participants wore four inertial measurement units (IMUs) providing both accelerometer and gyroscope assessments. The IMUs were connected via Bluetooth to the laptop and attached to participants’ wrists and ankles with Velcro straps. Figure 1 shows the training set up. Participants trained alone in the laboratory at ETH Hönggerberg (Zurich, Switzerland) wearing comfortable sports clothes and shoes. Two postgraduate students supported the participants and systematically observed them throughout the intervention. Furthermore, they ensured that the training principles of optimal load and progression were present [57, 58]. Training intensity was individually adapted to target a moderate to vigorous training level [59, 60]. Intervention characteristics as frequency, duration and training intensity were based on recommendations for fall prevention in elderly [44, 59–61] and on studies showing positive training effects of exergame training in older adults [62].

### Primary outcome

Usability of the newly developed exergame prototype was evaluated using quantitative and qualitative assessments. A mixed method approach was chosen similar to other studies which evaluated the usability of exergames [35]. Questionnaires were completed by participants after three training sessions (between-measurement) and after the intervention period (post-measurement).

#### Questionnaires

The System Usability Scale (SUS) includes 10 items rated on a 5-point Likert scale (0 = “strongly disagree” to 4 = “strongly agree”) and is a validated and reliable scale for evaluating subjective usability of newly developed devices and systems [63, 64]. The sum of all item scores was multiplied with 2.5 and led to the SUS score ranging between 0 to 100, whereas higher scores indicate better usability [63]. Based on the verbal categorization rate of Bangor [65], we expected a SUS score ≥ 70 for an “acceptable system”. An additional question was added at the end of the SUS, asking participants about their general opinion of the Active@Home exergame. This question was also rated on a 5-point Likert scale (0 = “I don’t like it” to 4 = “I like it a lot”) and the mean was calculated over all participants.

The Game Experience Questionnaire (GEQ) assessed several categories of subjective game experience (competence, immersion, flow, tension, challenge, negative affect, positive affect) [31, 66], and includes in total 42 items rated on a 5-point Likert scale (0 = “not at all” to 4 = “extremely”). *Competence* implies feelings of being successful, strong or skilful in the game. *Immersion* includes the interest and pleasure of a player in the game. *Flow* summarizes the feelings of being deeply concentrated and absorbed, forgetting time and losing connection to the world outside the game. *Tension* includes feelings of annoyance, frustration and pressure. *Challenge* implies feelings of being stimulated and challenged. *Negative affect* summarizes feelings related to a bad mood and boredom, whereas *positive affect* includes feeling of happiness and enjoyment. The GEQ was analyzed by calculating the average rating for each of the seven categories [67]. Two categories involved only negative coded items (tension and negative affect). These two categories were reverse evaluated [66].

#### Training observation and feedback

The usability protocol was structured in six categories: (1) functionality and interaction with the system, (2) IMUs, (3) design, (4) training principles, (5) exercises, and (6) emotions. It was filled in by the supervisors observing the participants during their training sessions. The participants were requested to “think aloud” and mention all thoughts that came to their mind while using the exergame [68]. Furthermore, the protocol included general feedback from participants. The collected observations and statements were separated in positive and negative aspects for each category.

#### Training compliance

An attendance protocol, filled in by the supervisors after each training session, was used to record the number of visited training sessions. The adherence rate was calculated using the number of visited training sessions as percentage of the maximum possible training sessions [36, 69]. A 70% attendance rate (15 visited out of 21 total training sessions planned) was considered “being adherent” to the training program [69, 70]. For attrition, the number of participants lost during the trial was recorded (drop-outs) and calculated as a percentage of the total sample size. Considering the median rate for attrition in fall prevention interventions for clinical trials, a 10% attrition rate (two drop-outs) was regarded acceptable [70]. Drop-outs were not considered in the calculation of the adherence rate. Reasons for non-adherence and drop-outs were, when given by the participants, recorded with a special interest in mal-compliance related to usability issues.

### Secondary outcomes

As secondary outcomes, physical and cognitive functions as well as cortical activity were measured before and after the intervention period (pre-measurement and post-measurement, respectively).

#### Physical functions

Parameters of gait kinematics were assessed using the Physilog5 IMU (Gait Up Sàrl, Lausanne, Switzerland), which has been shown to reliably measure gait performance [71]. The Physilog5 IMUs were fixed to the top of the right and left forefoot of participants using elastic straps. A USB port allows data transfer to the computer for further data analysis. A walking protocol involving at least 50 gait cycles was used [72]. Participants walked a straight distance of 80 m under two conditions: (1) single-task condition (ST): participants were instructed to walk at preferred speed without talking; (2) dual-task condition (DT): participants had to walk at preferred speed and simultaneously count backwards (cognitive task) in steps of seven from a randomly given number between 200 and 250. In this condition, participants were asked to perform both tasks concurrently and not to prioritise one task at the cost of the other. This is a common method to measure multitasking capabilities [73, 74]. Two walking steps for initiation and termination were discarded in order to analyse steady state walking [75]. Speed [m/s], cadence [steps/min], stride length [m], and minimal toe clearance [cm] were evaluated and expressed as mean values of both legs in the two walking conditions. For each parameter, the dual-task cost (DTC) of walking was calculated as a percentage of loss of the DT relative to the ST condition according to the formula: DTC [%] = (ST – DT)/ST × 100 [76].

To assess lower extremity functioning, the Short Physical Performance Battery (SPPB) was applied [77, 78]. A maximum of 12 points can be achieved where a low score is associated with a higher risk of falls [79]. The SPPB includes a balance test, a 4 m-walk test and a 5-chair rises test (maximally 4 points for each subtests). Details about the protocol can be found elsewhere [78]. In line with Eggenberger et al. [73], we extended the balance test with two additional tasks to avoid ceiling effects. The first additional task was a 20s single-leg stance (with preferred leg) where two points were achieved for reaching 20s, one point for 10–20s and zero points for <10s. The second additional task was a single-leg stance with eyes closed (with preferred leg) where one point was assigned for every 5 s of successful task achievement. For the extended version of the SPPB, the maximum point score is unlimited. For the analysis, the total score of the extended SPPB was calculated as well as the score for each subtest (balance score, 4 m-gait score, 5-chair rises score). For pre- and post-measurement comparison, time measures of the 4 m-gait test and the 5-chair rises test were also evaluated.

#### Cognitive functions

Higher cognitive functions such as working memory, divided and selective attention, inhibition and mental flexibility were assessed using four tests of the computerized test battery Test of Attentional Performance (TAP, D-TAP 2.3 VL, PSYTEST, Psychologische Testsysteme, Herzogenrath, Germany). The TAP is a valid assessment of different attentional and executive functions [80]. The following tests were performed on a computer using two answer buttons: *Working memory* (difficulty level 3), *Divided attention*, *GoNogo* (1 out of 2), *Set-shifting* (alternating letters and numbers). Each test was preceded by a short familiarization session. Details about this protocol can be found elsewhere [80]. For each of the four tests, reaction times [ms], number of errors and omissions were analysed.

#### Cortical activity and analysis

In order to assess cortical oscillatory activity, 5 minutes of resting state electroencephalography (EEG) were recorded at 500 Hz sampling rate, using a 20-channels dry-electrodes cap (ENOBIO 20, Neuroelectrics, Barcelona, Spain) placed according to the international 10–20 system [81] and referenced using the Driven-Right-Leg (DRL) / Common Mode Sense (CMS) technique (two external electrodes placed on either side of the left earlobe with an ear-clip). Before electrode placement on the forehead and earlobe, the skin was prepared with abrasive paste (H + H Medizinprodukte GbR, Münster, Germany).

EEG data analysis was performed using custom scripts written in MATLAB R2017b (The Mathworks, Natick, Massachusetts, USA) and using the EEGLAB 14.1.0b open source toolbox [82]. EEG data was first high-pass filtered [zero-phase Hamming windowed sinc FIR, cut-off frequency (− 6 dB) 0.5 Hz, passband edge 1 Hz, transition bandwidth 1 Hz, order 1651] and subsequently low-pass filtered [zero-phase Hamming windowed sinc FIR, cut-off frequency (− 6 dB) 45 Hz, passband edge 40 Hz, transition bandwidth 10 Hz, order 167]. Further analysis was performed to seven parieto-occipital EEG electrodes (Pz, P3/4, P7/8, and O1/2) only, since this cortical area is widely used to detect individual alpha frequency (IAF) reliably [83, 84]. Channel rejection was performed using the automatic procedure supplied by the *clean_rawdata* EEGLAB extension by taking into account if the correlation of a channel to a reconstruction of it based on other channels, in a given time window, was less than 0.4 as well as if a channel was flat for more than 5 seconds. On average, ~ 95% of the parieto-occipital channels in the pre-measurement EEG recordings remained for further analysis (σ: ~ 10%; range: ~ 71–100%) and ~ 92% (σ: ~ 9%; range: ~ 71–100%) in the post-measurement EEG recordings. Artefactual data points were rejected if their amplitude was higher than ±75 μV within a 500 ms width time window as detected by the *trimOutlier* EEGLAB plugin. On average, ~ 6% of data was rejected in the pre-measurement EEG recordings (σ: ~ 9%; range: ~ 0–30%) and ~ 8% (σ: ~ 14%; range: ~ 0–48%) in the post-measurement EEG recordings. Afterwards, two IAF measures were estimated: peak alpha frequency (PAF) and center of gravity (CoG), by means of the *restingIAF* v1.0 open source package available from https://github.com/corcorana/restingIAF. This allowed a fully automatic and reliable strategy to determine IAF estimates during resting state EEG recordings, of which a more detailed and extensive description can be found elsewhere [84, 85]. Briefly, one-sided channel-wise power spectral density (PSD) was first calculated in the 1-40 Hz frequency range by the Welch’s modified periodogram method, using a 2048 sample (~ 4 s) Hamming window (50% overlap) across segments (frequency resolution = 0.244 Hz) and normalized by dividing each PSD channel estimate (within the passband) by the mean spectral power. Then, each PSD estimate was smoothed using a Savitzky-Golay filter with frame length equals to 11 frequency bins and polynomial degree of five. From the smoothed PSD and within an a priori defined alpha frequency band (7-13 Hz), evident frequency peaks were detected and IAF estimates from spectral peaks’ boundaries were computed. Using the first derivative to detect spectral peaks seemingly yields true estimates compared to simply searching from maximal values within a predefined alpha frequency band [83]. Finally, IAF estimates were computed by averaging the obtained spectral peaks estimates across channels. The minimum number of valid channels necessary to estimate IAF was set to one, given the relatively low-density parieto-occipital EEG channels used for this analysis. Additionally, spectral power within the alpha frequency band was calculated by averaging in each participant the PSD estimates of all the included channels, and then summing the obtained channels mean power across the alpha frequency band. Alpha spectral bandwidth was defined as the individual PAF ±2 Hz.

### Other outcome measures

The participants wore a StepWatch (Orthocare Innovations LLC, Edmonds, Washington, USA) for 1 week before the intervention period started to assess their daily physical activity behaviour. The StepWatch recorded every step and calculated the number of steps for each day. The mean of 7 days was used as baseline characteristic. Furthermore, the participants rated their current training motivation on a Visual Analog Scale (1 = unmotivated lethargic smiley to 5 = motivated happy smiley) before every training. After each training session, the participants estimated their perceived exertion on the Borg scale from 6 to 20 (6 = “less than very light”, 20 = “more than very hard”) for Tai Chi-inspired and dance exercises, respectively.

### Statistical analysis

For all statistical analysis, SPSS 23.0 for Windows (SPSS Inc., Chicago, Illinois, USA) was used. Descriptive statistics were generated for all variables. Following a conservative approach and due to non-normality of some of the data, confirmed by both Shapiro-Wilk test and Q-Q-plots, non-parametric testing was applied. Intragroup differences between the two measurements were analysed by Wilcoxon signed-rank test. A significance level of α = 0.05 was applied. Correlational effect sizes (r), according to the following equation: r = z/√(n1 + n2) with n1 = n at pre-measurement and n2 = n at post-measurement, were calculated in MS Office Excel (version 2016) and reported according to Cohen [86]: an effect size of r = 0.10 indicates a small effect, r = 0.30 a medium effect, and r ≥ 0.50 a large effect. For pre- and post-measurement comparisons, drop-outs were excluded from analysis (per-protocol analysis). The analysis does not consider intention-to-treat analysis because of a clear description of the drop-out reasons [87]. Moreover, only participants who reached 70% of the maximal possible training sessions were included in the pre-post-comparison.

## Results

**Fig. 2:**
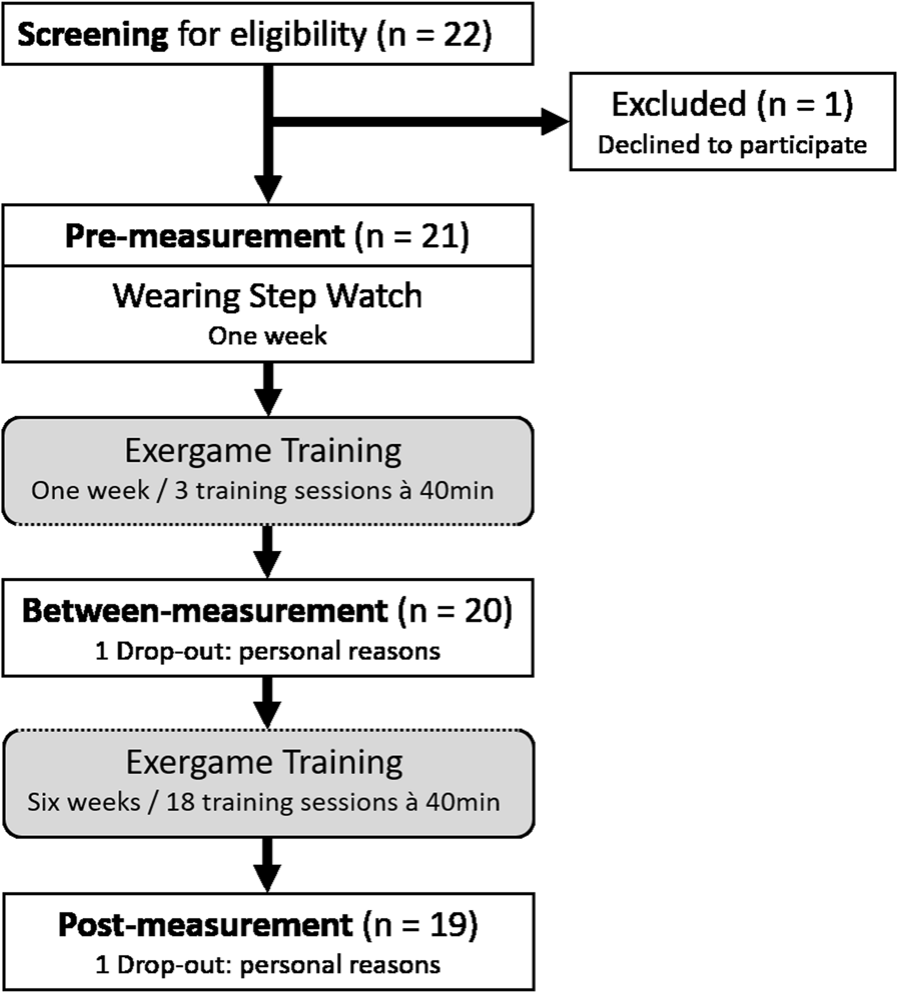
Study flow chart. Screening of participants for eligibility included an assessment of cognitive and health state. Physical and cognitive functions as well as brain activity were measured at pre- and post-measurement. Questionnaires assessing usability and game experience were filled in at between- and post-measurement

A total of 21 participants signed informed consent and were included in the study. Nineteen participants completed the seven-week training intervention. The two male, 71 years old and highly educated drop-outs were comparable to the rest of the sample regarding their characteristics. Table 1 summarizes the demographic characteristics and screening measures of the remaining participants. The study flow chart is presented in Fig. 2.

**Table 1:** Demographic characteristics and screeing values

Participant characteristics	*n* = 19
Age in years	71.4 ± 6.1 (65–91)
Weight [kg]	69.7 ± 19.5 (42–122)
Height [cm]	169.9 ± 8.8 (150–181)
BMI [kg/m^2^]	24.3 ± 5.0 (17–37)
Daily physical activity^a^	7410 ± 2079 (4605–12,247)
MOCA Score	28.1 ± 1.4 (26–30)
Female [n, %]	10 (52.6)
Education [n, %]	
Primary school	1 (5.3)
Upper school	0 (0.0)
Apprenticeship	9 (47.4)
Gymnasium	2 (10.5)
University	7 (36.8)
Fear of falling [n, %]	
Never	14 (73.7)
Sometimes	5 (26.3)
Often	0 (0.0)
Always	0 (0.0)
Number of falls during last month^b^ [n, %]	
Never	17 (89.5)
Once	2 (10.5)
More than once	0 (0.0)
Self-evaluation of health state [n, %]	
Very good	4 (21.1)
Good	14 (73.7)
Medium	1 (5.3)
Bad	0 (0.0)
Self-evaluation of balance [n, %]	
Very good	5 (26.3)
Good	8 (42.1)
Medium	6 (31.6)
Bad	0 (0.0)
Self-evaluation of muscle strength [n, %]	
Very good	0 (0.0)
Good	18 (94.7)
Medium	0 (0.0)
Bad	1 (5.3)

Data are mean values ± standard deviation (range) or number of participants per category (absolute and relative frequency) as indicated. *MoCA* Montreal Cognitive Assessment. ^a^Average steps per day measured with a StepWatch. ^b^Self-stated.

### Primary outcome results

The scores of the SUS and GEQ measured after the three initial training sessions (between-measurement) and after the training period (post-measurement) are presented in Table 2. The additional question, asking participants about their general opinion of the Active@Home exergame, showed a mean score (± SD) of 3.1 ± 0.7 (*n* = 20) at between-measurement and a mean score (± SD) of 3.0 ± 0.8 (*n* = 19) at post-measurement (on a scale from 0 = “I don’t like it” to 4 = “I like it a lot”). Table 3 summarizes the main feedback of the participants and observations of the supervisors during the training. No adverse events were recorded during the intervention period.

**Table 2:** Results of questionnaire ratings

Questionnaires	T2 (*n* = 20)	T3 (*n* = 19)	z	p	r
System Usability Scale (SUS)	75.0 (67.5; 87.5)	75.0 (70.0; 85.0)	−0.240	.823	0.04
Game Experience Quesionnaire (GEQ)					
Competence	2.5 (2.2; 2.7)	2.3 (2.2; 3.0)	−0.081	.945	0.01
Immersion	1.9 (1.5; 2.5)	2.3 (1.5; 2.7)	−0.881	.395	0.14
Flow	1.3 (0.7; 1.8)	1.0 (0.7; 1.5)	−1.281	.210	0.21
Tension	0.2 (0.0; 0.5)	0.2 (0.0; 0.3)	−1.279	.229	0.21
Challenge	1.2 (1.0; 1.5)	1.2 (0.7; 1.5)	−1.455	.157	0.24
Negative Affect	0.2 (0.0; 0.6)	0.5 (0.2; 0.8)	−3.134	.001*	0.51
Positive Affect	2.8 (2.4; 3.2)	2.8 (2.0; 3.3)	−0.569	.590	0.09

Data are median values (interquartile range). T2 = after three training sessions (between-measuement), T3 = after training intervention (post-measurement). *p < .05, p-values are exact sig. Two-tailed. T2-T3 differences were evaluated using Wilcoxon signed-rank test (n = 19). An effect size of r = 0.10 indicates a small effect, r = 0.30 a medium effect, and r ≥ 0.50 a large effect [86]. SUS scale ranges from 0 to 100. GEQ scale ranges from 0 = “not at all” to 4 = “extremly”. Two categories of the GEQ (tension, negative affect) have to be evaluated reversely which means a low score is favorable.

**Table 3:** Summary of usability protocol with supervisors’ observations and participants’ feedback

Criteria	Positive aspects	Negative aspects
Functionality and interaction with the system	− Good and stable connection of laptop (with system software) to TV^a^	− Technical issues (as system crashes or frozen pictures on the screen)^a,b^
	− Simple set up^b^	− Unstable (Bluetooth) connection of IMUs to the system^a,b^
	− Easy usable game composition^b^	− Navigation via laptop keyboard instead by IMUs (as cursors)^a^
		− Inaccurate evaluation of movements by IMUs (evaluation algorithms)^a^
IMUs	− Comfortable to wear (participants did not notice them during training)^b^	− Suboptimal material of IMU cover (cover expanded after heating up while charging)^a^
		− Suboptimal material of IMU fixation strap (difficult to clean the Velcro fixation, material sticks to some clothes)^a,b^
		− Difficulties to attach the IMUs with the Velcro fixation (especially at wrists)^a,b^
Design	− Exciting game story of travelling around Europe to different cities^b^	− No variation in music^b^
	− Pleasant music^b^	− No explanation about feedback system (colour code, performance score)^b^
	− Helpful cues (arrows) to prepare the next movement^b^	
	− Virtual instructor guiding through exercises^b^	
	− Helpful indication of number of exercise repetitions^b^	
Training principles	− Visual feedback with colour code (green, orange, red) during exercising^b^	− No specific feedback regarding exercise execution and single body part movements^a,b^
	− Performance score as feedback after exercising^b^	− Training load and progression determined by supervisors (no automatic progression)^a^
		− Low variability in exercises^a,b^
		− Training load even in high levels not exhausting^1,2^
Exercises	− Clear structure of exercise levels^b^	− No proper introduction of exercises (just start copying the movements of the virtual instructor)^a,b^
	− Complex exercises with additional arm movements provide more fun than simple (boring) movements^b^	− Only frontal view of exercises (side view missing)^b^
		− No further information about exercise (e.g. muscles involved)^b^
Emotions	− General enjoyment and fun^a,b^	− Frustrated and displeased by technical issues and inaccurate evaluation of movements^a,b^
	− Increased motivation through virtual instructor (better than train alone)^b^	− Missing challenge due to easy exercises^a,b^
	− Happy when seeing a progress or achieving higher performance score^a,b^	− Bored of low training variability^a,b^

*IMU* Inertial measurement unit. ^a^supervisors’ observations, ^b^participants’ feedback.

Participants completed on average 18 out of the 21 total training sessions, resulting in an adherence rate of 90.0%. Six participants reached the recommended training dose of three training sessions per week. Reasons for non-adherence were: holidays, being busy and family affairs. Considering the two drop-outs, the attrition rate amounted to 9.5%. One drop-out reason was related to a lack of training motivation and the other reason was related to personal issues.

### Secondary outcome results

On group level, the gait speed under the dual-task walking condition increased significantly (z = − 2.012, *p* = .045, r = 0.33) after the training intervention. In the 5-chair rises test of the SPPB, the time needed to perform five chair rises significantly decreased (z = − 2.853, *p* = .003, r = 0.46) after exergame training. In the divided attention task of the TAP, participants reacted significantly faster (z = − 2.495, *p* = .011, r = 0.40) to auditory stimuli after the training intervention. No significant changes in resting state EEG were found for the pre- and post-measurement comparison. The results are presented in Table 4.

**Table 4:** Results of pre- and post-measurements of physical and cognitive functions and cortical acitivty

	Assessed by	Pre (T1)	Post (T3)	z	p	r
Physical functions	Gait analysis					
	Speed [m/s]					
	ST walking	1.42 (1.36; 1.61)	1.52 (1.36; 1.62)	−0.684	.515	0.11
	DT walking	1.26 (1.17; 1.46)	1.31 (1.22; 1.51)	−2.012	.045*	0.33
	DT costs in %	6.4 (5.1; 17.6)	8.4 (3.0; 18.2)	−1.449	.156	0.24
	Cadence [steps/min]					
	ST walking	117.2 (114.6;	120.7 (115.1; 126.1)	−1.529	.134	0.25
	DT walking	125.7)	113.2 (109.5; 120.4)	−1.690	.096	0.27
	DT costs in %	111.7 (105.4; 116.9) 4.0 (1.8; 8.7)	3.5 (1.0; 9.0)	−1.046	.312	0.17
	Stride length [m]					
	ST walking	1.48 (1.41; 1.56)	1.47 (1.41; 1.56)	−0.402	.709	0.07
	DT walking	1.36 (1.28; 1.50)	1.36 (1.33; 1.49)	−1.610	.113	0.26
	DT costs in %	3.8 (2.1; 10.3)	4.2 (0.0; 9.2)	−1.006	.332	0.16
	Minimal toe clearance [cm]					
	ST walking	2.3 (2.1; 3.1)	2.9 (2.0; 3.4)	−1.891	.060	0.31
	DT walking	2.2 (1.7; 2.7)	2.7 (1.6; 3.2)	−1.248	.225	0.20
	DT costs in %	3.9 (−4.9; 16.6)	6.7 (−7.1; 19.3)	−0.241	.829	0.04
	Extended SPPB					
	Total score	14 (13; 15)	14 (13; 15)	−0.266	.797	0.04
	Balance score	6 (6; 8)	7 (5; 7)	−0.134	.947	0.02
	4 m-gait score	4 (4; 4)	4 (4; 4)	0.000	1.000	< 0.01
	4 m-gait time [s]	3.3 (2.9; 3.7)	3.2 (2.8; 3.6)	−1.449	.153	0.24
	5-chair rises score	4 (3; 4)	4 (3; 4)	−0.816	.750	0.13
	5-chair rises time [s]	10.5 (8.3; 12.8)	8.8 (7.3; 12.3)	−2.853	.003*	0.46
Cognitive functions	Test of Attentional Performance					
	Working memory					
	RT [ms]	741 (597; 843)	677 (603; 840)	−0.348	.742	0.06
	Errors	3 (0; 6)	3 (1; 5)	−0.416	.696	0.07
	Omissions	3 (2;4)	3 (1; 4)	−1.719	.088	0.28
	Divided attention					
	RT auditory [ms]	652 (584; 769)	594 (580; 714)	−2.495	.011*	0.40
	RT visual [ms]	893 (822; 948)	881 (834; 945)	−0.080	.945	0.01
	Errors	1 (0; 3)	1 (0; 2)	−0.641	.541	0.10
	Omissions	1 (0; 3)	1 (1; 2)	−1.388	.190	0.23
	Selective attention					
	RT [ms]	454 (397; 487)	468 (396; 504)	−0.543	.602	0.09
	Errors	1 (0; 2)	0 (0; 2)	−0.265	.848	0.04
	Omissions	0 (0; 0)	0 (0; 0)	−1.890	.125	0.31
	Mental flexibility					
	RT [ms]	932 (798; 1124)	848 (786; 1018)	−1.610	.113	0.26
	Errors	3 (1; 9)	3 (1; 4)	−1.163	.258	0.19
Cortical activity	Resting state EEG					
	Peak alpha frequency [Hz]	9.3 (8.4; 9.9)	9.3 (8.7; 10.0)	−1.274	.232	0.26
	Center of gravity [Hz]	9.2 (8.5; 9.7)	9.3 (8.4; 9.6)	−1.013	.340	0.20
	Alpha spectral power [μV^2^]	20.5 (16.1; 35.7)	27.0 (16.8; 46.9)	−0.078	.970	0.02

Data are median values (interquartile range). n = 19. *p < .05, p-values are exact sig. Two-tailed. Pre-post differences were evaluated using Wilcoxon signed-rank test. An effect size of r = 0.10 indicates a small effect, r = 0.30 a medium effect, and r ≥ 0.50 a large effect [86]. *ST* single-task, *DT* dual-task. DT costs are calculated as (ST – DT)/ST × 100. *SPPB* Short Physical Performance Battery. *RT*  reaction time

### Other outcome results

The participants’ average daily physical activity resulted in 7410 steps per day (Table 1). Training motivation was on average rated with 4.5 ± 0.5 by participants (on a visual scale from 1 = unmotivated lethargic smiley to 5 = motivated happy smiley). The average rating of participants’ perceived exertion for Tai Chi-inspired exercises was 10.7 ± 2.3 and for dance exercises 9.7 ± 2.0 on the 20-point Borg scale (6 = “less than very light” to 20 = “more than very hard”).

## Discussion

The primary aim of this study was to assess the usability of the newly developed Active@Home exergame prototype in older adults and to provide implications for further development. Furthermore, potential training-related changes in physical and cognitive functioning were explored. In general, the newly developed exergame seems usable for healthy older adults and the study results indicate possible positive influences on physical and cognitive functions. However, the later have to be interpreted with caution due to the lack of a control group.

### Usability of the exergame training

An acceptable usability of the Active@Home exergame prototype was evident in the questionnaire ratings with a SUS score of 75/100 after three training sessions as well as at the end of the intervention. This result is in line with previous studies showing that exergames are in general well accepted and usable for older adults, especially when considering their needs [29, 35, 64]. However, some limitations were evident in the observations of supervisors during training and in the feedback of participants. Most limitations were related to technical issues that occurred during training with the exergame prototpye (e.g. system crashes or unstable connection to IMUs). Considering that older adults’ technical knowledge and experiences with new technologies are often restricted, a newly developed technology-based system has to work without any technical failures. Moreover, a simple set-up, stable connections, and an intuitive interaction with the system are mandatory aspects especially when the training system is expected to be deployed at older adults’ home. The importance of an age-appropriate design and flawless technical functionality were emphasized to be crucial for the usability of exergames [64, 88, 89]. Otherwise, technological problems can lead to unintentional avoidance and disuse of a training system.

Even though some technical issues occurred during training with the exergame prototype, training compliance in this study was high. The adherence rate of 90.0% indicates a good acceptance of the training system. Moreover, an attrition rate of less than 10% (9.5% in this study) can be considered acceptable. Interestingly, the reasons for non-adherence were not related to the usability of the exergame. Only one participant prematurely terminated the intervention due to a lack of motivation. However, the general training motivation was rated high (on average 4.5/5). High motivation is an important key factor for long-term exercising that, in turn, leads to lasting training effects [90, 91]. In accordance with our results, adherence has been shown to be often higher in interventions using exergames compared to standard fall prevention exercises [36, 69, 92]. An explanation might be the entertaining and captivating character of exergames resulting in positive emotions [64, 66, 93]. Positive emotional experiences, in turn, might increase training motivation and therefore, as mentioned before, enhance long-term compliance [94]. Regarding emotions in this study, the GEQ scores showed medium to high ratings of positive emotions, e.g. feeling captivated and pleased, as well as low ratings of negative emotions, e.g. feeling tensed and annoyed, both after three training sessions and at the end of the intervention. The significant increase in negative affect from between- to post-measurement might be due to an accumulation of negative emotions related to technical issues. Nevertheless, we can conclude that the Active@Home exergame provided an overall positive emotional experience which might have led to the high training motivation and the high training compliance found in this study.

In order to maximize the benefits of a training, exercise interventions should implement some basic training principles including feedback, optimal load, progression and variability [57]. The supervisors’ observations and participants’ statements showed that the feedback system integrated in the Active@Home exergame was, currently, not yet optimal. To facilitate learning and the acquisition of new skills and knowledge, optimal feedback is mandatory [95]. Negative feedback helps to correct errors while positive feedback satisfies and often increases motivation [95]. One reason for the dissatisfaction with the feedback system might be that the movement evaluation was slightly inaccurate and, thus, the evaluation algorithms have to be improved. Another reason might be that the implemented feedback system was too superficial (e.g. evaluation of whole-body movements), while more specific and precise feedback was missing (e.g. feedback of upper/lower limbs’ movements separately). Nevertheless, participants could benefit from the real-time feedback while training and were proud and motivated when reaching high performance scores. Considering the training principles of optimal load and progression, the exercise demands have to be continuously adapted to each participant’s skill level achieving a challenging situation and avoiding an under- or overload. For the Active@Home exergame intervention, however, some participants stated that the training was not challenging enough. A reason might be that the exergame had to comply with a wide range of ability levels of older adults. Therefore, more difficult and complex exercises should be added to the exergame to satisfy also fitter older adults. Despite these limitations, participants generally enjoyed the simple system set-up, the game story of travelling around Europe, the music during the exercises and the guidance of the virtual instructor.

To summarize, this study showed a useable exergame prototype with several points that should be considered for further improvement of the system: technical aspects, movement evaluation and implementation of training principles. After this first evaluation of the newly developed exergame prototype in a monitored and supervised environment, the findings warrant an extended trial testing the feasibility and usability of the adjusted Active@Home exergame in an in-home setting.

### Exploration of potential effects of the exergame training

Our study showed significant improvement of dual-task walking speed after exergame training, which is in line with the results of several other studies [74, 96, 97]. Exergaming is discussed to train dual-task abilities, which are important in daily life activities and for fall prevention in older adults [98]. Furthermore, pre-post comparisons revealed a significant increase of functional lower limbs muscle power in this study. This improvement might be related to the Tai Chi-inspired exercises embedded in the exergame training. These exercises were mainly performed in squat position placing load on the lower limb and core muscles [99–101]. Despite of this potential improvement in muscle strength, the training guidelines considered for strength training turned out to be unsuitable [44]: The required intensity of 70–79% of 1RM (one repetition maximum) could not be reached using mainly body weight-based exercises. Furthermore, the breaks in between strength exercises, composed for very large exercise efforts, were too long. Consequently, the participants’ subjective rating of perceived exertion for Tai Chi-inspired training was on average on a low rate (10.7/20). The optimal zone for strength training corresponds to Borg scale ratings of 15–17 on the 20-point scale [102, 103]. The suboptimal training load and the rather short training period of 7 weeks might have restricted the training impact on additional physical outcomes. We, therefore, recommend to increase exercise complexity and intensity and extend the training period.

In the computer-based cognitive tests, study results showed a significantly improved reaction time for auditory stimuli in a divided attention task in pre-post comparisons. Surprisingly, the reaction time to visual stimuli did not improve. This could be related to the fact that especially for dance exercises listening to auditory information (music, rhythms) was important. These findings are similar to the results of a recent exergame study in older adults [74]. Furthermore, several studies including a combined physical-cognitive training as exergaming provided indications that this training approach boosts particular executive functions as mental flexibility, inhibition, or working memory [96, 104–106]. Executive functions are higher order cognitive functions playing an important role in guiding through everyday life. The absence of improvements in executive functions in this study might be due to the fact that the cognitive training itself was not specific enough or too short.

For older adults, a variety of studies reported age-related changes in cortical oscillatory activity [107]. Most of these studies showed a general “slowing” of the resting state EEG with a power increase in the slow frequency ranges (< 7 Hz) and a power decrease in higher frequencies (e.g. alpha power frequency band: ~ 7-13 Hz) especially in posterior brain regions [13, 14]. Accordingly, the individual alpha frequency peak is known to decrease in the later part of lifespan [107]. Furthermore, alpha power is considered to be positively correlated to global cognitive status in healthy and impaired older adults [107–110]. The pathological processes on neuronal level during aging leading to altered EEG power are presumed to be counteracted with physical and cognitive training [14]. However, we found no significant changes in resting state EEG in pre-post comparisons in our study, which might be also due to the short intervention period.

## Limitations

As this is a pilot study with primary focus on usability outcomes, secondary results have to be interpreted with caution especially because of small sample size and missing control group [111, 112]. To carefully evaluate the effectiveness of the Active@Home exergame, a further larger study (randomized controlled trial) will be conducted. Moreover, the training period should be extended to increase the potential for training improvements. Regarding the relatively high physical fitness level of the participants, caution has to be provided by generalizing these results to the overall older adults. To ensure high acceptability, newly developed training systems and interventions must be developed according to the target users’ needs, attitudes, and expectations. One reason for the abovementioned result in this study of general high usability might be that the development and design of the Active@Home exergame has followed a user-centered design approach. Older adults as target users have been involved from the beginning in the processes of training and game development. Nevertheless, a discrepancy exists between the high usability ratings in the questionnaires compared to the limitations observed by the supervisors and stated by the participants during training. This “positive bias” in the quantitative methods might be due to a general enthusiasm of the participants for the basic idea of the training approach, their pleasure to exercise despite of the potential for improvement of the training system, their satisfaction to support research or due to social aspects.

### Implications

Based on the results of this usability study, several implications can be provided to support the development and improvement of exergame training approaches for older adults:

- A mature concept should be composed including game design, technology and training aspects.
- The system set-up should be simple and age-appropriate (e.g. regarding technological devices, screen size, game design, in-program navigation).
- If technological devices as IMUs are attached to players’ bodies, material and fixation must be optimized, user-friendly, comfortable and size-adjustable.
- Easy applicable charging solutions should be used for technological devices as IMUs.
- Players should be sufficiently informed about the game story, goals, evaluation and feedback system.
- Exercises have to be well explained and instructed before and during their execution (e.g. exercise tutorial with frontal and side view, further information about exercise goals, cues during execution, and time indications).
- Including music in exergames might be important and motivating provided that the music is appropriate and fits the game story, training content and movements.
- Performance feedback has to be accurate, easy to understand and as detailed as possible, since feedback is one of the most important motivational factors and necessary for training benefits.
- Games and exercises should be challenging for a wide range of player prerequisites.
- Automatic configuration of optimal training load and progression is desirable.
- A high variability in games and exercise options is needed.

## Conclusion

Our study results showed a general high usability of the newly developed Active@Home exergame with high training compliance and positive emotional game experience in older adults. The results of this study provide some important suggestions for further exergame development and other technology-based training systems for older adults. Implications include elimination of technical issues as well as ensuring a simple system set-up, accurate feedback and challenging and diverse exercises for a wide range of skill levels. Furthermore, the study results provide some indications that this multicomponent video game-based training might enhance physical and cognitive functions in older adults. The study results warrant further development of the Active@Home exergame and may help other researchers in the design process of exergame interventions for older adults.

## Data Availability

All data generated and analysed during this study are included in this published article.

## Abbreviations

DT: Dual-task
DTC: Dual-task cost(s)
EEG: Electroencephalography
GEQ: Game experience Questionnaire
IMU: Inertial Measurement Unit
MOCA: Montreal Cognitive Assessment
SPPB: Short Physical Performance Battery
ST: Single-task
SUS: System Usability Scale
TAP: Test of Attentional Performance
